# Musculoskeletal manifestations of lymphoma: a case series

**DOI:** 10.1259/bjrcr.20210182

**Published:** 2022-09-12

**Authors:** David Jackson, Anthony G Ryan, Elaine Walsh, Mark Bolger, Brian Hennessy, Ezzat ElHassadi, Senthil Kumar, Joan Heneghan, Donal O'Driscoll

**Affiliations:** 1 Department of Medicine, University Hospital Waterford, Dunmore Road, Waterford, Ireland; 2 Department of Radiology, University Hospital Waterford, Dunmore Road, Waterford, Ireland; 3 Department of Haematology, University Hospital Waterford, Dunmore Road, Waterford, Ireland

## Abstract

Lymphomas are a heterogenous group of cancers of the lymphatic system in which disease primarily arises in lymph nodes. Extranodal disease is common; however, musculoskeletal involvement is rare. Imaging plays an important role in the diagnosis and staging of all lymphomas. In this case series, we present examples of musculoskeletal involvement of lymphoma encountered at our institution. We outline the clinical presentation and imaging findings of each and use these cases to review the features that can help to differentiate lymphoma from other musculoskeletal lesions.

## Introduction

Lymphomas are cancers of the lymphatic system, accounting for approximately 2% of all cancer diagnoses each year and are ranked the sixth most common cancer type in Ireland.^1,2^ They are a heterogenous group of more than 50 subtypes which are broadly categorised into Hodgkin and Non-Hodgkin lymphoma (NHL), with NHL accounting for the vast majority (80–90%). Disease primarily arises in lymph nodes but can occur in nearly any tissue, with extranodal disease present in approximately 25–40% of cases.^3,4^ Musculoskeletal involvement is rare and is usually the result of secondary spread but may also represent primary extranodal disease.^4–7^

In this case series, we present seven cases of biopsy-proven lymphoma, each of whom had musculoskeletal lesions on initial imaging. We discuss in turn the imaging findings of each including radiographic, CT, MRI and positron emission tomography (PET). Finally, we review the typical imaging features of lymphoma in muscle and bone, and outline the characteristics that differentiate these from other malignant lesions within the musculoskeletal system.

### Case 1

**Figure 1.**
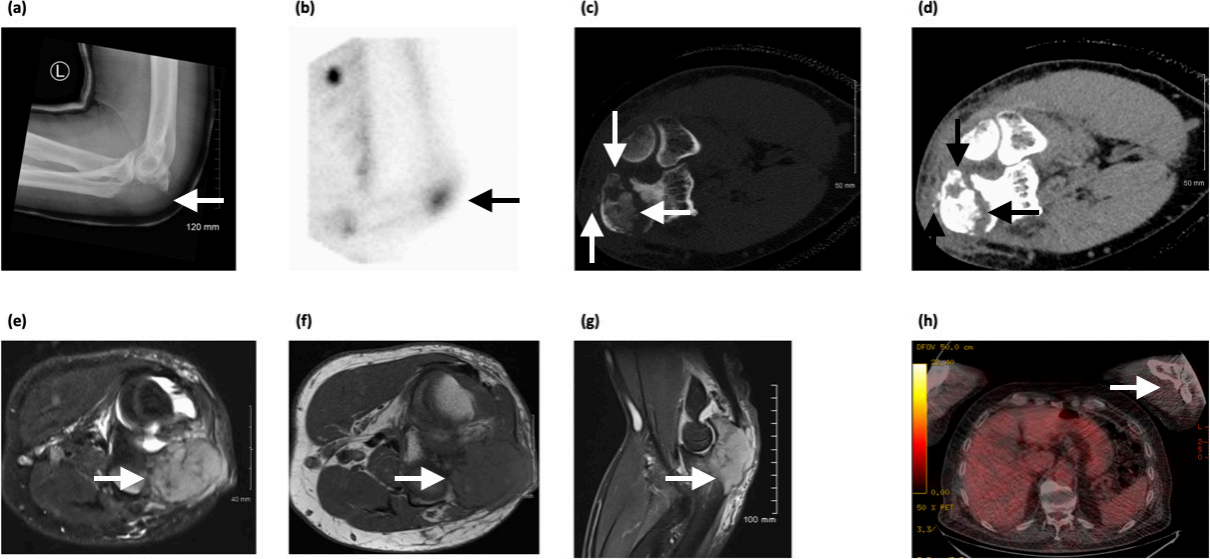
(**a**) Radiograph of left elbow demonstrating permeative destruction of the left olecranon, (**b**) bone scintigraphy showing increased radionuclide uptake at corresponding site, (**c, d**) axial CT image demonstrating typical features of musculoskeletal lymphoma including cortical destruction, extension into the soft tissue and pathological fracture of the olecranon with associated bony fragments, (**e**) axial *T*_2_ weighted fast spin echo MR image and (**f**) *T*_1_ weighted MR image in which the lesion is hyperintense (T2) and hypointense (T1) to surrounding muscle, (**g**) sagittal *T*_1_ weighted turbo inversion recovery magnitude MR image showing hyperintense lesion (arrow), (**h**) end-of-treatment PET-CT with no evidence of disease in the left elbow. PET, positron emission tomography.

A 57-year-old gentleman presented to the emergency department with a 1-week history of left elbow pain which he first noticed while playing golf. On examination, his left elbow was mildly swollen with tenderness at the posterior aspect of the olecranon. A radiograph revealed permeative destruction of the proximal ulna in the region of the olecranon process (Figure 1a). Bone scintigraphy showed increased radionuclide uptake corresponding to the identified lesion (Figure 1b). Non-contrast CT showed cortical destruction and extension into the adjacent soft tissue (Figure 1c). CT also showed a fracture of the left olecranon extending intra-articularly with punctate bony fragments (Figure 1d). MR imaging showed a destructive lesion of the olecranon which is hyperintense on *T*_2_ weighted imaging and hypointense on *T*_1_ weighted imaging relative to adjacent skeletal muscle (Figure 1 [e–g]). Histology confirmed diffuse large B-cell lymphoma. End-of-treatment PET-CT demonstrated no residual disease in the elbow (Figure 1h).

### Case 2

**Figure 2.**
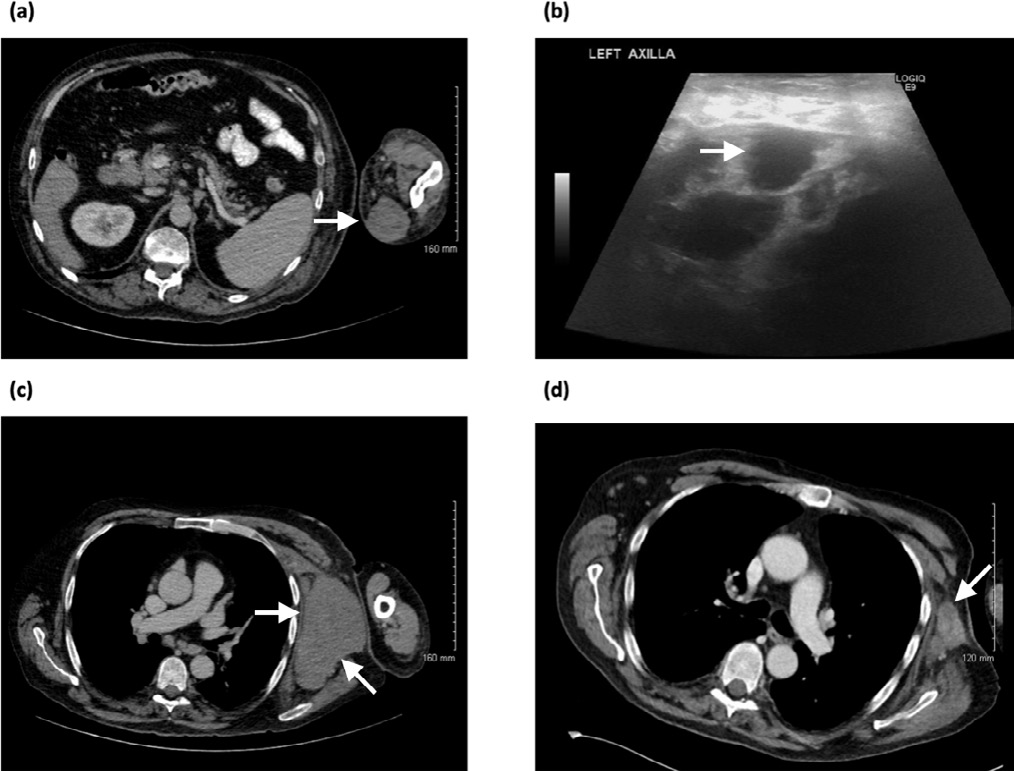
(**a**) Axial CT image showing a large well-defined soft tissue mass in the medial aspect of the left elbow measuring 5 × 4 cm, superficial and separate from the underlying musculature, (**b**) sonogram of the left axilla showing an enlarged hypoechoic pathological lymph node, (**c**) axial CT image showing a large conglomerate nodal mass in the left axilla, (**d**) end-of-treatment axial CT image showing significant reduction in size of the axillary mass.

A 73-year-old gentleman presented with a 2-month history of a progressively enlarging mass on the medial aspect of his left elbow associated with pitting oedema. The patient had also noticed a new swelling in the left axilla. He denied any weight loss, fever or night sweats. CT confirmed a large subcutaneous mass in the medial aspect of the left elbow measuring 5 × 4 cm which was separate from the underlying musculature (Figure 2a). CT also revealed a large conglomerate nodal mass in the left axilla with abnormal submandibular, supraclavicular, mediastinal, hilar, mesenteric, para-aortic, iliac and inguinal lymph nodes (Figure 2c). Splenomegaly was also noted. Ultrasound of the axilla revealed multiple enlarged pathological lymph nodes (Figure 2b). Histology confirmed diffuse large B-cell lymphoma. End-of-treatment CT showed significant reduction in size of both the axillary mass (Figure 2d) and left elbow mass (image not available).

### Case 3

**Figure 3.**
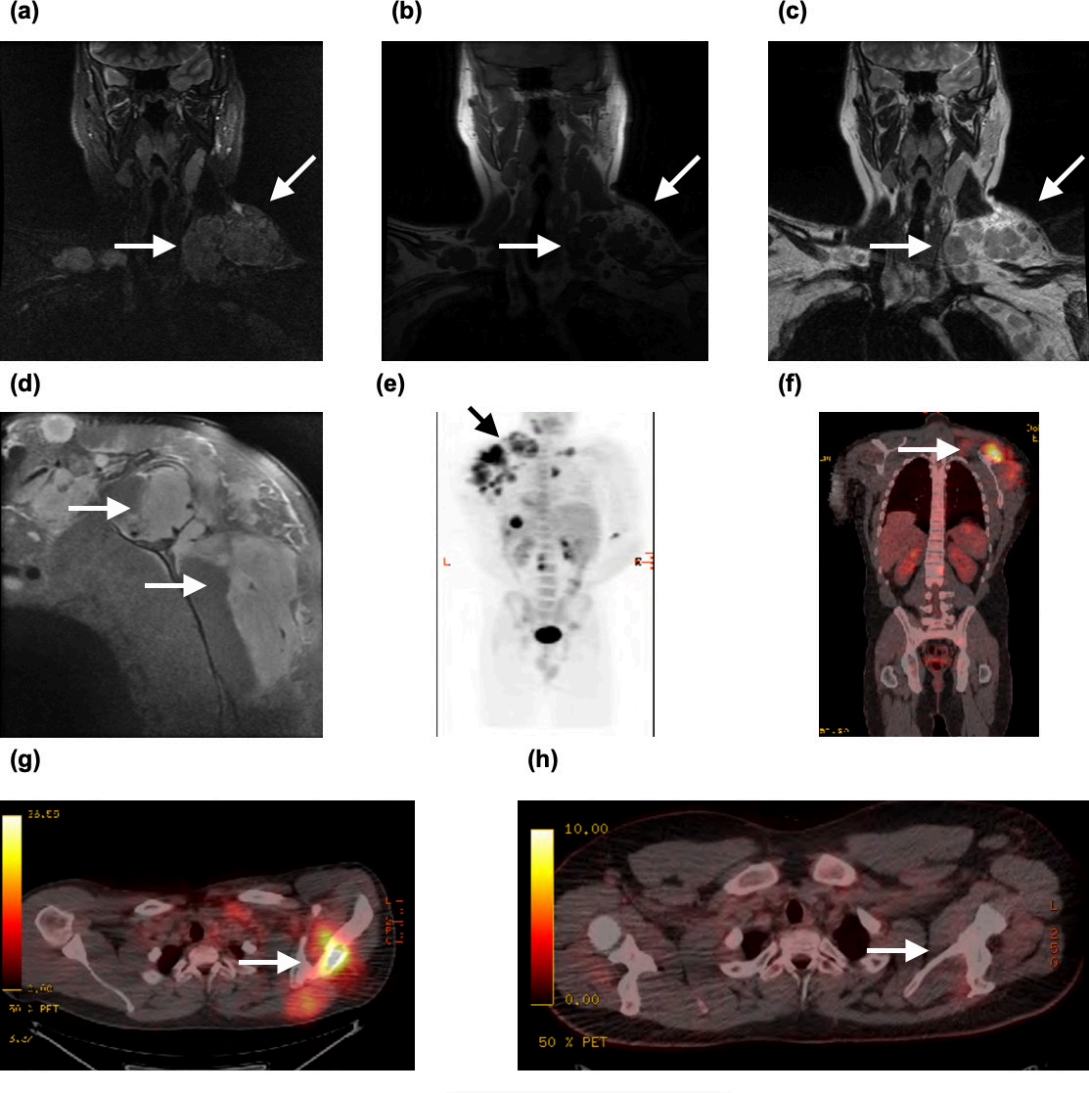
(**a**) Coronal STIR MR image of the left brachial plexus and shoulder showing a large volume conglomerate nodal mass with mixed density, (**b**) coronal *T*_1_ weighted MR image showing multiple enlarged lymph nodes which are isointense to skeletal muscle and hypointense to surrounding fat, (**c**) corresponding coronal *T*_2_ weighted MR image of the lymph nodes demonstrating intermediate intensity. (**d**) Sagittal fat-supressed MR image of the left shoulder showing enlarged hyperintense supraspinatus and infraspinatus muscles, (**e, f, g**) PET-CT image with corresponding FDG-avid lesions in the left shoulder/axilla and (**h**) end-of-treatment PET demonstrating complete resolution of disease. FDG, fludeoxyglucose; PET, positron emission tomography; STIR, short-tau inversion recovery.

A 23-year-old male developed pain and swelling of his left neck/supraclavicular region following a workplace accident in which his arm had become trapped in heavy machinery. The swelling progressed over a period of 2 months causing significant restriction of left shoulder movement. On examination, there was marked supraclavicular and axillary lymphadenopathy, with further swelling of the pectoral and scapular region. MRI left shoulder and brachial plexus (Figure 3 [a–d]) showed large volume pathological supraclavicular and axillary nodes bilaterally, more prominent on the left, as well as enlargement of both the infraspinatus and supraspinatus muscles with abnormal signal, consistent with a probable lymphoproliferative disorder. PET-CT (Figure 3 [e–g]) revealed intensely fludeoxyglucose (FDG)-avid large volume lymphadenopathy predominantly in the left supraclavicular region. In addition, there was evidence of osseous destruction and periosteal thickening of the left acromion process and upper scapula with high intensity uptake of the supraspinatus and infraspinatus muscles. Biopsy confirmed Hodgkins lymphoma. End-of-treatment PET (Figure 3h) demonstrated complete resolution of previously identified FDG-avid lymphoma.

### Case 4

A 62-year-old gentleman presented with a 5-month history of progressively enlarging right posterior thigh lesion associated with weight loss and generalised lethargy. On examination, a firm, non-tender palpable mass measuring approximately 10 cm in diameter was easily identified in the posterior compartment of the right thigh.

**Figure 4.**
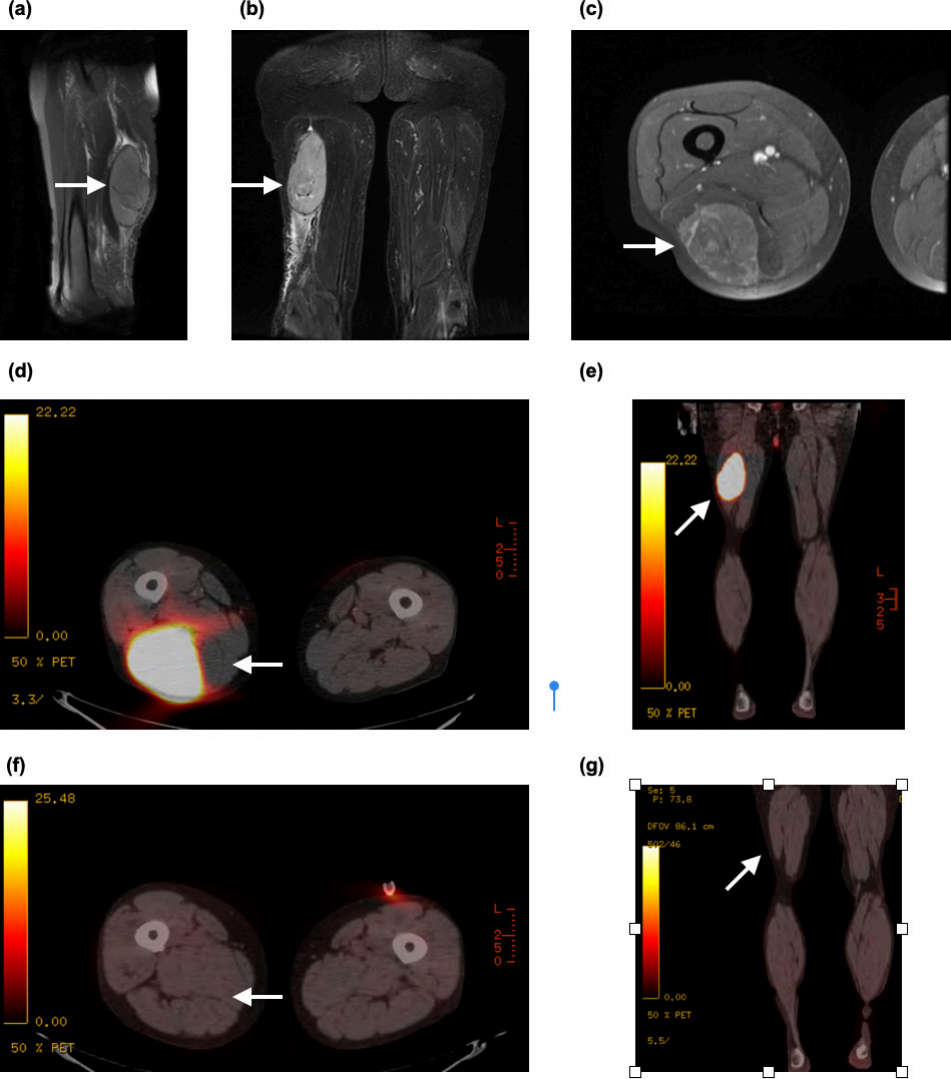
(**a**) Sagittal *T*_2_ weighted MR image showing a large well-circumscribed mass in the posterior compartment of the right thigh which demonstrates intermediate intensity relative to skeletal muscle, (**b**) coronal STIR MR image, (**c**) axial *T*_1_ weighted MR image of the same lesion demonstrating contrast enhancement and displacement of adjacent skeletal muscle, (**d, e**) axial and coronal PET-CT showing intense FDG uptake and (**f, g**) corresponding end-of-treatment scans showing complete resolution. PET, positron emission tomography; STIR, short-tau inversion recovery.

MRI showed a 12 × 6 × 5 cm, well-defined mass in the posterior compartment (Figure 4 [a–c]). The mass demonstrated contrast enhancement with displacement of the adjacent muscle tissue including the semimembranous and long head of the biceps femoris. PET-CT (Figure 4 [d–e]) demonstrated a grossly enlarged right biceps femoris with intense FDG uptake. Histology confirmed diffuse large B-cell lymphoma. End-of-treatment PET-CT (Figure 4 [f–g]) showed complete resolution of the thigh lesion.

### Case 5

**Figure 5.**
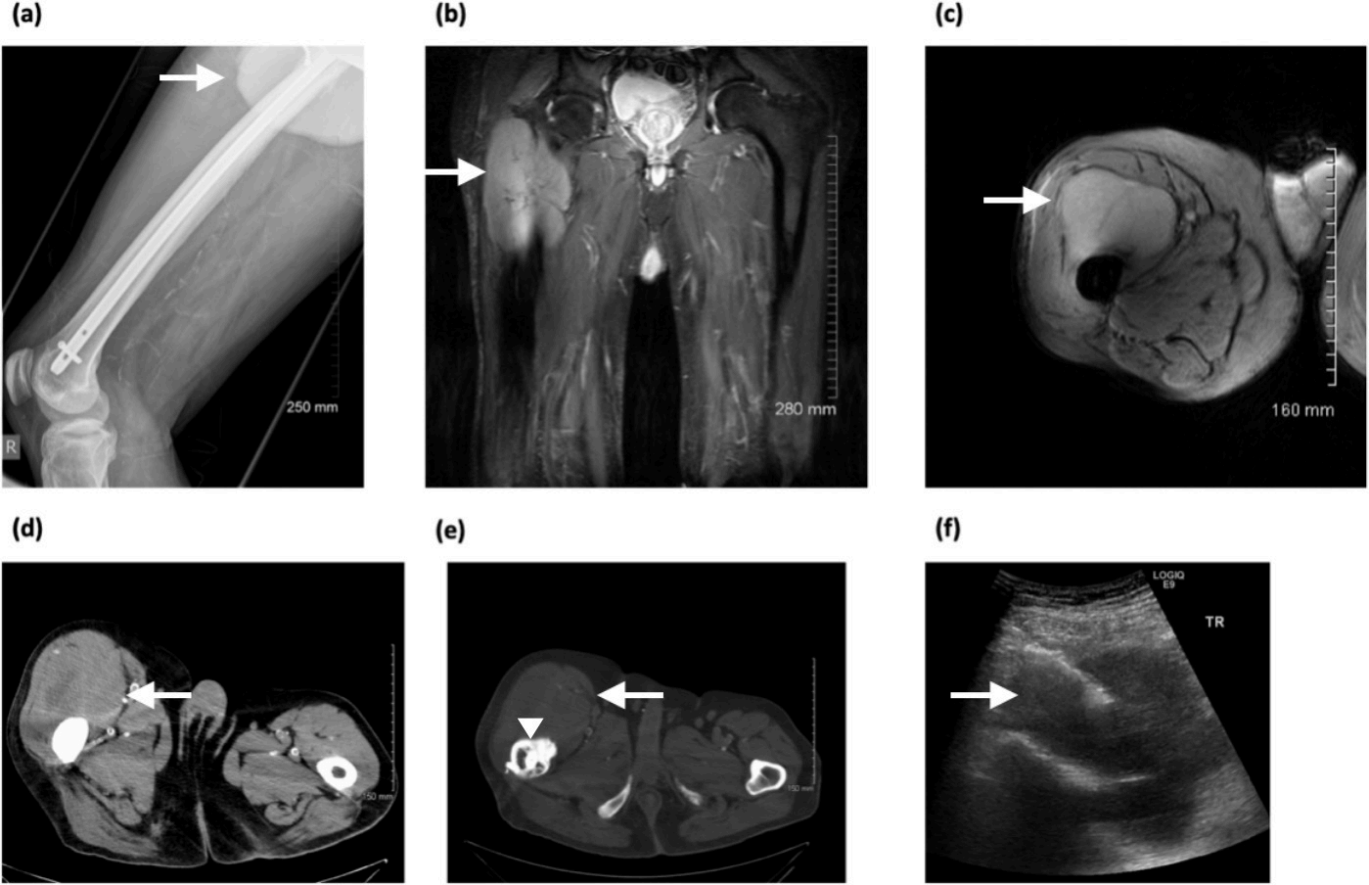
(**a**) Lateral radiograph of right femur showing an area of increased density in the upper right thigh, (**b, c**) coronal and axial *T*_2_ weighted MR images showing large 18 × 6 cm hyperintense mass in upper lateral thigh which is well defined and non-infiltrative, (**d, e**) axial CT images showing the mass (arrows) and periosteal reaction (arrow head), (**f**) sonogram during biopsy of the mass.

A 74-year-old male, with a history of right femoral nailing 2 years prior, presented with swelling of the right thigh. Plain radiograph demonstrated increased soft tissue density in the upper right thigh (Figure 5a). MRI showed a large soft tissue mass 18 × 6 cm anterior to the right femur (Figure 5 [b and c]). The mass was hyperintense relative to adjacent skeletal muscle and well-defined without any evidence of bony destruction or infiltration. CT demonstrated associated cortical reaction, destruction and sclerotic changes of the proximal anterior femur (Figure 5 [d and e]). Ultrasound-guided biopsy was performed which confirmed diffuse large B-cell lymphoma (Figure 5f). CT-TAP demonstrated retroperitoneal lymphadenopathy. The patient completed three cycles of chemotherapy; however, an interim CT revealed disease progression and the patient was referred for palliative radiotherapy.

### Case 6

**Figure 6.**
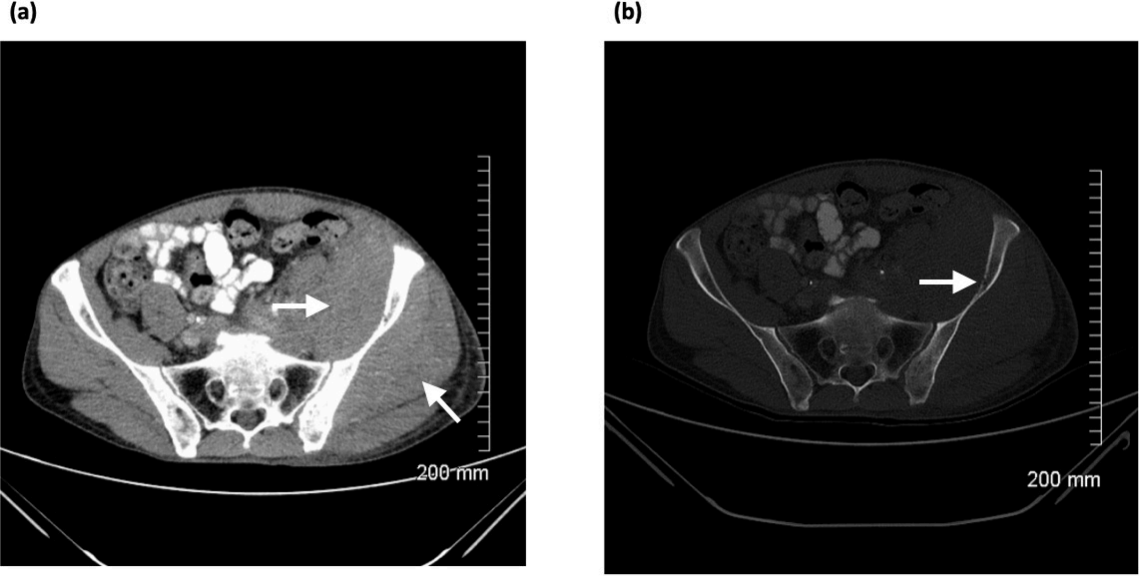
(**a**) Axial CT image showing large soft tissue mass infiltrating the left iliopsoas and gluteal muscle which is isodense to adjacent musculature, (**b**) axial CT image of same lesion with bony window demonstrating subtle permeative destruction of the left iliac bone.

A 55-year-old male presented with a history of left lower limb swelling, abdominal pain and reduced appetite. CT showed a large soft tissue mass infiltrating the iliopsoas and gluteal muscles with associated permeative destruction of the left iliac bone (Figure 6 [a and b]). Ultrasound revealed abnormal psoas and iliac muscles as well as necrotic left inguinal nodes (images not available). Biopsies confirmed diffuse large B-cell lymphoma. End-of-treatment PET-CT demonstrated complete resolution of disease (images not available).

### Case 7

**Figure 7.**
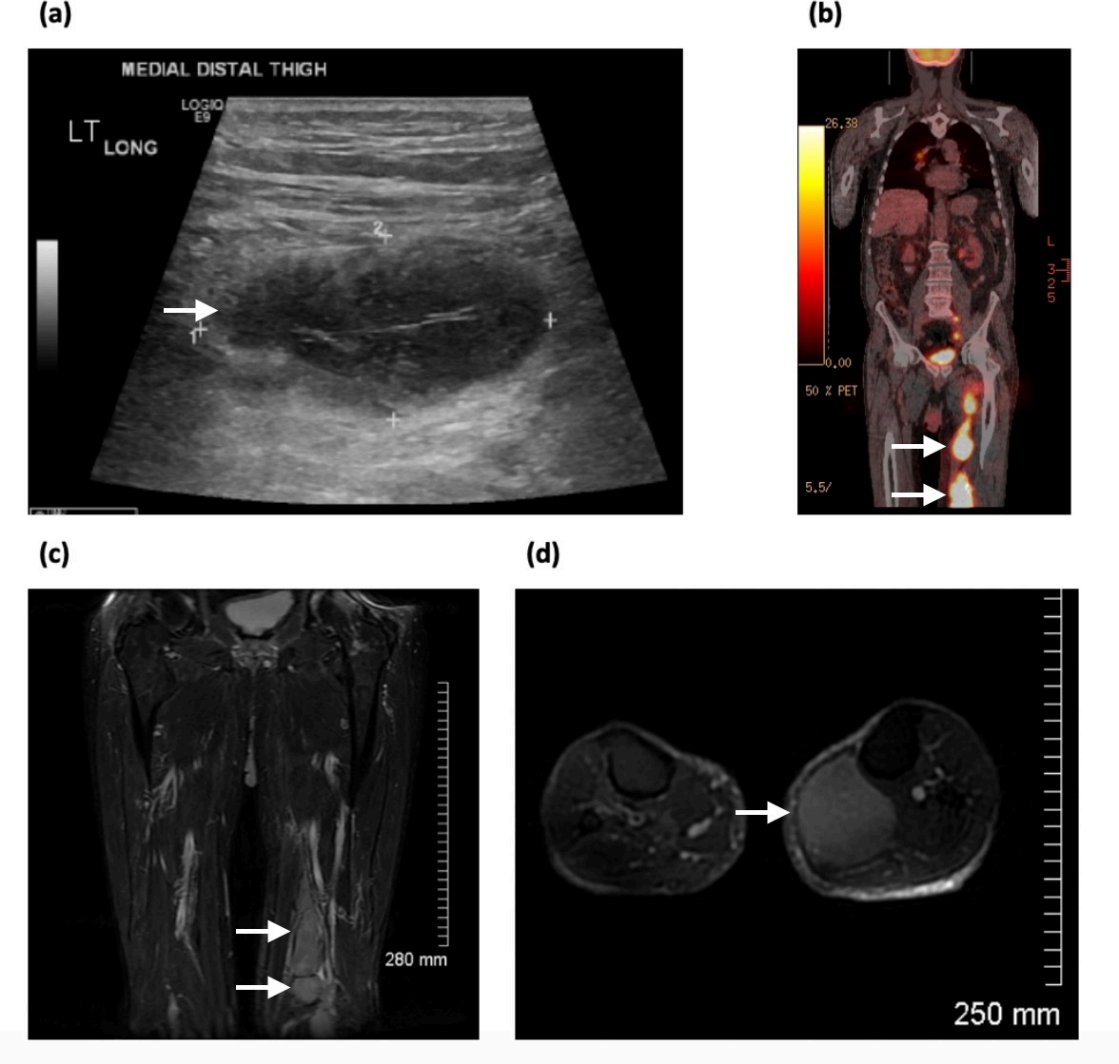
(**a**) Sonogram demonstrating hypoechoic poorly defined lesion, (**b**) PET-CT image showing multiple FDG-avid lesions in the medial aspect of the lower limb, (**c**) coronal *T*_2_ weighted MR image showing multiple hyperintense lesions, (**d**) axial *T*_2_ weighted MR image demonstrating hyperintensity of the lesions with well-defined margins. FDG, fludeoxyglucose; PET, positron emission tomography.

An 84-year-old gentleman, who had previously received treatment for testicular non-Hodgkins lymphoma and had achieved complete remission, presented to the Emergency Department 6 months post-treatment with swelling of his left thigh. Doppler ultrasound was performed which showed no evidence of DVT; however, two non-specific lesions were identified: one inside the musculature of the medial distal thigh measuring 4.8 × 2.2 cm, and a second well-circumscribed hypodense nodule measuring 3.5 × 1.4 cm in the subcutaneous area of the right suprapatellar region (Figure 7a). In addition, the superior medial gastrocnemius muscle was reported abnormal with a hypoechoic appearance. Biopsies of this region were acquired. MRI revealed multiple T2 hyperintense lesions throughout the left lower limb both intramuscular, and coursing along the left femoral artery and vein (Figure 7[c and d]).

PET-CT demonstrated multiple FDG avid lesions above and below the diaphragm, with bulky disease in the left lower limb, primarily within the left gastrocnemius muscle (Figure 7b). Biopsies obtained confirmed diffuse large B-cell lymphoma. Due to the extent of disease and clinical deterioration the patient was not suitable for further chemotherapy.

## Discussion

Imaging plays an important role in the diagnosis, staging and treatment of lymphoma. Typically, more than one imaging modality is required to fully and accurately evaluate lymphoma of the musculoskeletal system. This may include radiography, CT, MRI, bone scintigraphy and/or PET-CT.

Lymphoma of bone, sometimes termed osseous lymphoma, accounts for 5% of extranodal lymphomas and represents 7% of all bone malignancies.^8^ Osseous lymphoma can be subclassified into primary bone lymphoma (PBL), multifocal PBL, or disseminated lymphoma with secondary osseous involvement that is either (a) within or (b) greater than 6 months from initial diagnosis. Diffuse large B-cell lymphoma accounts for the majority of osseous lymphomas.^9–11^ Clinical presentation includes bone pain (61%), soft tissue swelling (31%), pathological fracture (22%) and typical B-symptoms (13%), although these are rare in PBL.^12^ PBL and multifocal PBL typically affect the appendicular skeleton, with multifocal PBL occasionally involving the axial skeleton.^10^ Secondary osseous lymphoma preferentially affects the axial skeleton.^13^

Radiographic features of osseous lymphoma are variable and non-specific. Patterns can be normal, lytic, sclerotic or mixed lytic/sclerotic.^5^ A permeative or “moth-eaten” appearance is the most common.^9^ This appearance can also be seen with infection, Ewing sarcoma, osteosarcoma, multiple myeloma and metastases from other malignancies. Features that can indicate advanced local disease include cortical destruction, periosteal reaction and extraosseous soft tissue masses.^9^ Periosteal reaction patterns can be linear, lamellated or disrupted. Sclerotic lesions can occur, most frequently with Hodgkins lymphoma, however, lytic lesions remain the most frequently encountered in all subtypes.^13,14^ The ivory vertebra sign representing diffuse sclerosis of a vertebral body is a radiological sign associated with osseous lymphoma but can also be seen in a number of conditions including osteoblastic metastases from prostate and breast cancer as well as Paget disease and osteosarcoma.^15^

Bone scintigraphy is useful for the detection of multifocal osseous involvement however findings are non-specific.^10,15^ CT provides a more detailed assessment of osseous lymphoma, with better visualisation of cortical and trabecular destruction, periosteal reaction and extraosseous extension. One particular feature on CT that is suggestive of lymphoma is the relative absence of cortical destruction which is more commonly seen with osteosarcoma.^16^

MR imaging in osseous lymphoma is particularly useful for assessing marrow involvement.^17^ Abnormal marrow generally exhibits low intensity on *T*_1_ weighted images and high intensity on *T*_2_ weighted images. This, however, is not specific for lymphoma and low signal intensity on *T*_2_ weighted images can be seen.^17,18^ MR imaging is also useful in evaluating extraosseous soft tissue masses which again demonstrate low signal intensity on *T*_1_ weighted images and high signal intensity on *T*_2_ weighted images.^9^

Muscular lymphoma accounts for 1.4% of all lymphomas and similarly, this can be due to disseminated lymphoma, local extension or primary muscular lymphoma.^19,20^ As with osseous lymphoma, patients may present with localised soft tissue swelling, pain and/or B-symptoms.^21^ The thigh and upper arm are the most common sites of disease which may be a focal mass or diffuse infiltration of the muscle.^21–23^ Ultrasonography, while non-specific and of limited use for staging, does play an important role in evaluating a solid mass/enlarged muscle, particularly for guiding biopsies. There are no specific sonographic features of muscular lymphoma, however, soft tissue lesions are usually solid, hypoechoic and can have either well-defined or poorly defined borders.^21^ CT findings for muscle involvement are non-specific. Muscle enlargement can be seen, with the abnormal tissue often isodense to normal muscle and enhancement post-contrast is variable.^24,25^ CT is, however, useful for identifying extent of disease and bony involvement/destruction. MR imaging is the most useful imaging modality for the assessment of muscular lymphoma. Either a discrete mass, or diffuse muscle infiltration may be seen, with the abnormal tissue isointense on *T*_1_ weighted images or hyperintense on *T_2_* weighted images to surrounding muscle tissue.^6^ Contrast enhancement can be diffuse homogenous or heterogenous exhibiting peripheral thick band-like enhancement or marginal septal enhancement. Features that are suggestive of skeletal muscle lymphoma include muscle enlargement with long segmental or multicompartmental involvement, deep fascial involvement, skin thickening, subcutaneous stranding and traversing vessels.^26^

## Conclusion

Lymphoma represents a significant proportion of malignancies each year and timely identification is crucial given the often favourable outcomes if diagnosed early. Extranodal disease is common and can occur in almost any tissue. Bone and muscle involvement is rare, yet remains an important differential for musculoskeletal lesions as these can be the only presenting symptom. Imaging findings are often non-specific; however, certain imaging features, outlined in this case review, can help to characterise such lesions.

## Learning points

1. Lymphoma is an important differential to consider for new bony/muscular lesions, either as primary musculoskeletal lymphoma or secondary to disseminated disease.
2. Radiological diagnosis is challenging due to the heterogeneity of the disease and multiple modalities should be employed.
3. For osseous involvement, radiography and CT are useful for determining the pattern of destruction and extent of extraosseous spread, while MR imaging can demonstrate marrow involvement.
4. In muscular lymphoma, MR imaging is the most useful modality. Muscle enlargement with multicompartmental involvement and traversing vessels are features suggestive of lymphoma.

## References

[1] Zinzani PL. Lymphoma: diagnosis, staging, natural history, and treatment strategies. Semin Oncol 2005; 32: S4-10. doi: 10.1053/j.seminoncol.2005.01.008 15786020

[2] National Cancer Registry Ireland. Available from: https://www.ncri.ie/factsheets/non-hodgkins-lymphoma

[3] Rademaker J. Hodgkin’s and non-hodgkin’s lymphomas. Radiol Clin North Am 2007; 45: 69–83. doi: 10.1016/j.rcl.2006.10.006 17157624

[4] Freeman C, Berg JW, Cutler SJ. Occurrence and prognosis of extranodal lymphomas. Cancer 1972; 29: 252–60. doi: 10.1002/1097-0142(197201)29:1<252::aid-cncr2820290138>3.0.co;2-# 5007387

[5] Lim CY, Ong KO. Imaging of musculoskeletal lymphoma. Cancer Imaging 2013; 13: 448–57. doi: 10.1102/1470-7330.2013.0036 24334414 PMC3864222

[6] Chun CW, Jee WH, Park HJ, Kim YJ, Park JM, Lee SH, et al. MRI features of skeletal muscle lymphoma. AJR Am J Roentgenol 2010; 195: 1355–60. 10.2214/AJR.09.3904 21098195

[7] Binici DNR, Karaman A, Timur O, Tasar PNT, Sanibas AV. Primary skeletal muscle lymphoma: A case report. Mol Clin Oncol 2018; 8: 80–82. doi: 10.3892/mco.2017.1483 29387400 PMC5768100

[8] Fletcher C, Unni K, Mertens F. Pathology and genetics of tumours of soft tissue and bone: World Health Organization classification of tumours. Lyon, France: International Agency for Research on Cancer; 2002.

[9] Krishnan A, Shirkhoda A, Tehranzadeh J, Armin AR, Irwin R, Les K. Primary bone lymphoma: radiographic-MR imaging correlation. Radiographics 2003; 23: 1371–83. doi: 10.1148/rg.236025056 14615550

[10] Melamed JW, Martinez S, Hoffman CJ. Imaging of primary multifocal osseous lymphoma. Skeletal Radiol 1997; 26: 35–41. doi: 10.1007/s002560050188 9040141

[11] Ostrowski ML, Unni KK, Banks PM, Shives TC, Evans RG, O’Connell MJ, et al. Malignant lymphoma of bone. Cancer 1986; 58: 2646–55. doi: 10.1002/1097-0142(19861215)58:12<2646::aid-cncr2820581217>3.0.co;2-u 3779614

[12] Beal K, Allen L, Yahalom J. Primary bone lymphoma: treatment results and prognostic factors with long-term follow-up of 82 patients. Cancer 2006; 106: 2652–56. doi: 10.1002/cncr.21930 16700039

[13] O’Neill J, Finlay K, Jurriaans E, Friedman L. Radiological manifestations of skeletal lymphoma. Curr Probl Diagn Radiol 2009; 38: 228–36. doi: 10.1067/j.cpradiol.2008.07.001 19632500

[14] Ostrowski ML, Inwards CY, Strickler JG, Witzig TE, Wenger DE, Unni KK. Osseous hodgkin disease. Cancer 1999; 85: 1166–78. doi: 10.1002/(sici)1097-0142(19990301)85:5<1166::aid-cncr22>3.0.co;2-v 10091803

[15] Mulligan ME, McRae GA, Murphey MD. Imaging features of primary lymphoma of bone. AJR Am J Roentgenol 1999; 173: 1691–97. doi: 10.2214/ajr.173.6.10584821 10584821

[16] Hwang S. Imaging of lymphoma of the musculoskeletal system. Radiol Clin North Am 2008; 46: 379–96. doi: 10.1016/j.rcl.2008.03.008 18619386

[17] Hermann G, Klein MJ, Abdelwahab IF, Kenan S. MRI appearance of primary non-hodgkin’s lymphoma of bone. Skeletal Radiol 1997; 26: 629–32. doi: 10.1007/s002560050300 9428068

[18] Heyning FH, Kroon HMJA, Hogendoorn PCW, Taminiau AHM, van der Woude H-J. MR imaging characteristics in primary lymphoma of bone with emphasis on non-aggressive appearance. Skeletal Radiol 2007; 36: 937–44. doi: 10.1007/s00256-007-0335-1 17558503

[19] Komatsuda M, Nagao T, Arimori S. An autopsy case of malignant lymphoma associated with remarkable infiltration in skeletal muscles (author’s transl). Rinsho Ketsueki 1981; 22: 891–95. 7334615

[20] Lee VS, Martinez S, Coleman RE. Primary muscle lymphoma: clinical and imaging findings. Radiology 1997; 203: 237–44. doi: 10.1148/radiology.203.1.9122401 9122401

[21] Beggs I. Primary muscle lymphoma. Clin Radiol 1997; 52: 203–12. doi: 10.1016/s0009-9260(97)80274-7 9091255

[22] Samuel LM, White J, Lessells AM, Roddie H, Matheson LM. Primary non-hodgkins lymphoma of muscle. Clin Oncol (R Coll Radiol) 1999; 11: 49–51. doi: 10.1053/clon.1999.9009 10194587

[23] Lanham GR, Weiss SW, Enzinger FM. Malignant lymphoma. A study of 75 cases presenting in soft tissue. Am J Surg Pathol 1989; 13: 1–10. 2909193

[24] Malloy PC, Fishman EK, Magid D. Lymphoma of bone, muscle, and skin: CT findings. AJR Am J Roentgenol 1992; 159: 805–9. doi: 10.2214/ajr.159.4.1529847 1529847

[25] Panicek DM, Lautin JL, Schwartz LH, Castellino RA. Non-hodgkin lymphoma in skeletal muscle manifesting as homogeneous masses with CT attenuation similar to muscle. Skeletal Radiol 1997; 26: 633–35. doi: 10.1007/s002560050301 9428069

[26] Suresh S, Saifuddin A, O’Donnell P. Lymphoma presenting as a musculoskeletal soft tissue mass: MRI findings in 24 cases. Eur Radiol 2008; 18: 2628–34. doi: 10.1007/s00330-008-1014-x 18493781

